# Cell-free HPV-DNA as a high-accuracy biomarker for treatment de-escalation in HPV-positive head and neck squamous cell carcinoma

**DOI:** 10.3389/fonc.2025.1569877

**Published:** 2025-09-18

**Authors:** Alex Hollander, Taichiro Nonaka

**Affiliations:** ^1^ School of Medicine, Louisiana State University Health Shreveport, Shreveport, LA, United States; ^2^ Department of Cellular Biology and Anatomy, Louisiana State University Health Sciences Center, Shreveport, LA, United States; ^3^ Feist-Weiller Cancer Center, Louisiana State University Health Shreveport, Shreveport, LA, United States

**Keywords:** head and neck cancer, biomarker, liquid biopsy, cell-free DNA, circulating tumor DNA

## Abstract

**Objective:**

Head and neck squamous cell carcinoma (HNSCC) remains a devastating disease with significant morbidity and mortality, despite advances in treatment. HPV-positive HNSCC, in particular, has been increasing in incidence worldwide, yet optimal management strategies remain an unmet need. While patients with HPV-positive tumors have a better prognosis and improved response to therapy compared to HPV-negative cases, the long-term toxicities associated with standard treatments necessitate a shift toward treatment de-escalation strategies. However, the lack of biomarkers to guide patient selection for de-intensified therapy remains a critical challenge. We hypothesize that cell-free HPV-DNA (cfHPV-DNA) demonstrates high diagnostic accuracy and can serve as an effective non-invasive biomarker for early detection, disease monitoring, and treatment de-escalation in HPV-positive HNSCC. This meta-analysis aims to establish the clinical utility of cfHPV-DNA in diagnosing HNSCC and its potential role in guiding de-escalation strategies.

**Methods:**

A comprehensive literature search was conducted using PubMed, Web of Science, and Wiley to identify studies evaluating the diagnostic value of cfHPV-DNA in HNSCC. The population included HPV-positive HNSCC patients, the intervention was cfHPV-DNA detection via liquid biopsy, and the outcome was to assess the diagnostic performance of cfHPV-DNA. The pooled diagnostic parameters were analyzed using STATA and Revman.

**Results:**

Twelve studies involving 626 patients were included. The pooled sensitivity and specificity of cfHPV-DNA were 0.89 (95% CI: 0.71 – 0.96) and 0.99 (95% CI: 0.91 – 1.00), respectively. The positive and negative likelihood ratios were 66.55 (95% CI: 8.9 – 497.6) and 0.12 (95% CI: 0.04 – 0.33), with a pooled diagnostic odds ratio of 574.73 (95% CI: 55 – 6019). The area under the curve (AUC) was 0.98 (95% CI: 0.96 – 0.99), indicating exceptional diagnostic performance.

**Conclusion:**

The high diagnostic accuracy of cfHPV-DNA supports its potential as a valuable biomarker for early detection and risk stratification in HPV-positive HNSCC. Our findings suggest that cfHPV-DNA could provide a real-time, non-invasive tool to monitor treatment response and disease progression, allowing for personalized de-escalation approaches. Furthermore, we discuss the necessary steps toward FDA approval, emphasizing the need for standardized detection methods and large-scale validation studies to facilitate its integration into clinical practice.

## 1 Introduction

Head and neck squamous cell carcinoma (HNSCC) is a malignant neoplasm arising from the mucosal epitheium of the upper aerodigestive tract, including the oral cavity, oropharynx, hypopharynx, and larynx (1). Pathologically, HNSCC is characterized by dysplastic epithelial proliferation, leading to invasive carcinoma with varying degrees of keratinization (2). The etiology of HNSCC is multifactorial, with tobacco and alcohol consumption being well-established risk factors (3). However, over the past few decades, the role of high-risk human papillomavirus (HPV), particularly HPV type 16 (HPV16), has been increasingly recognized as a major etiological factor in a subset of HNSCC, primarily affecting the oropharynx (4–6).

HPV-driven HNSCC represents a distinct subset of head and neck cancers, particularly affecting the tonsils and base of the tongue (7, 8). The prevalence of HPV-positive HNSCC has been rising in Western countries, correlating with changing sexual behaviors (9–11). It is estimated that over 70% of oropharyngeal squamous cell carcinomas (OPSCC) are now associated with HPV, with HPV16 accounting for the vast majority of cases (12). The mechanism of HPV-driven carcinogenesis involves viral oncogenes E6 and E7, which inactivate tumor suppressor proteins p53 and retinoblastoma protein (pRb), respectively, leading to uncontrolled cell proliferation (13). Unlike tobacco- and alcohol-induced carcinogenesis, which is characterized by a high mutational burden, HPV-driven oncogenesis involves fewer somatic mutations but significant dysregulation of cell cycle control and immune evasion (14–17).

HPV-positive and HPV-negative HNSCC exhibit distinct clinical, pathological, and molecular features, influencing prognosis and therapeutic strategies (18). Clinically, patients with HPV-positive HNSCC are typically younger, non-smokers, and present with small primary tumors but large, cystic nodal metastases (19, 20). In contrast, HPV-negative HNSCC is commonly associated with older age, significant smoking and alcohol history, and presents with locally aggressive tumors with early tissue invasion and perineural spread (21, 22). Pathologically, HPV-positive tumors are often non-keratinizing, basaloid in morphology, and demonstrate strong p16 immunohistochemical positivity, which serves as a surrogate marker for HPV infection (23, 24). HPV-negative tumors, on the other hand, are frequently keratinizing squamous cell carcinomas with prominent dyskeratosis and are p16-negative (23). On a molecular level, HPV-negative HNSCC harbors a high frequency of *TP53* mutations, *CDKN2A* deletions, and *EGFR* amplifications, whereas HPV-positive tumors typically exhibit wild-type *TP53*, intact *CDKN2A*, and high expression of viral oncogenes *E6* and *E7* (14). Due to the lack of widespread somatic mutations in HPV-positive cases, cell-free HPV-DNA (cfHPV-DNA) detection may be a more suitable biomarker than circulating tumor DNA (ctDNA) in this subgroup.

HPV-positive HNSCC is associated with a significantly better prognosis compared to HPV-negative HNSCC, regardless of disease stage (25, 26). Patients with HPV-positive tumors demonstrate superior response rates to radiotherapy and chemotherapy, with 5-year overall survival rates exceeding 80%, compared to approximately 50% in HPV-negative cases (27, 28). Consequently, treatment de-intensification strategies are being explored for HPV-positive patients to minimize long-term toxicities without compromising oncologic outcomes (29, 30). In contrast, HPV-negative HNSCC carries a worse prognosis due to its aggressive nature, high recurrence rates, and resistance to conventional therapies (31, 32). Given the higher somatic mutation burden in HPV-negative cases, ctDNA, which reflects tumor-derived somatic mutations, may be a more suitable liquid biopsy biomarker for detecting residual disease and monitoring treatment response.

The current standard for diagnosing HNSCC involves a combination of clinical examination, imaging (CT, MRI, PET-CT), and tissue biopsy (33). p16 immunohistochemistry and HPV DNA/RNA detection are routinely performed in oropharyngeal tumors to determine HPV status. However, these diagnostic approaches have several limitations. Tissue biopsy is invasive, sometimes challenging in difficult-to-access locations, and may not always provide a conclusive diagnosis in small or necrotic tumors (34). Imaging lacks specificity for distinguishing between benign and malignant lesions, particularly in post-treatment settings where fibrosis and inflammation may confound results (35–37). HPV testing is currently based on tumor tissue samples, requiring an invasive biopsy, which is not ideal for longitudinal monitoring or for patients in whom obtaining a sample is difficult.

Liquid biopsy is an emerging non-invasive diagnostic approach that allows the detection of tumor-derived nucleic acids in body fluids such as blood and saliva (38, 39). In the context of HNSCC, two key liquid biopsy biomarkers are being investigated. Circulating tumor DNA (ctDNA) is a fragment of tumor-derived DNA that carries somatic mutations and is widely used in cancers with high mutational burdens. Given that HPV-negative HNSCC exhibits a high number of somatic mutations, including *TP53* mutations and *CDKN2A* deletions, ctDNA may be particularly useful for monitoring tumor burden and treatment response in this subgroup (14, 40). In contrast, HPV-positive HNSCC is characterized by a low somatic mutation rate but high expression of viral oncogenes, making cfHPV-DNA an attractive biomarker for detecting HPV-driven tumors, monitoring response to therapy, and assessing minimal residual disease (41). Additionally, cfHPV-DNA testing could be instrumental in treatment de-escalation strategies, as patients with HPV-positive HNSCC often require less aggressive treatment than their HPV-negative counterparts (42).

Given the distinct molecular and clinical differences between HPV-positive and HPV-negative HNSCC, as well as the limitations of current diagnostic methods, there is a critical need to evaluate the role of cfHPV-DNA as a non-invasive biomarker. While previous studies have explored the feasibility of cfHPV-DNA detection, the diagnostic accuracy and clinical applicability of this approach remain unclear as cfHPV-DNA may also derive from HPV-associated anogenital cancers (43). Further comprehensive evaluation is required to assess its integration into existing diagnostic workflows, considering the evolving methodologies and emerging clinical data. In this study, we aim to consolidate the existing evidence, provide a comprehensive evaluation of cfHPV-DNA’s diagnostic potential, and clarify its role in improving the detection and management of HNSCC. By synthesizing data from multiple studies, this analysis seeks to establish a more robust foundation for future research and potential clinical implementation.

## 2 Materials and methods

### 2.1 Search strategy

**Figure 1:**
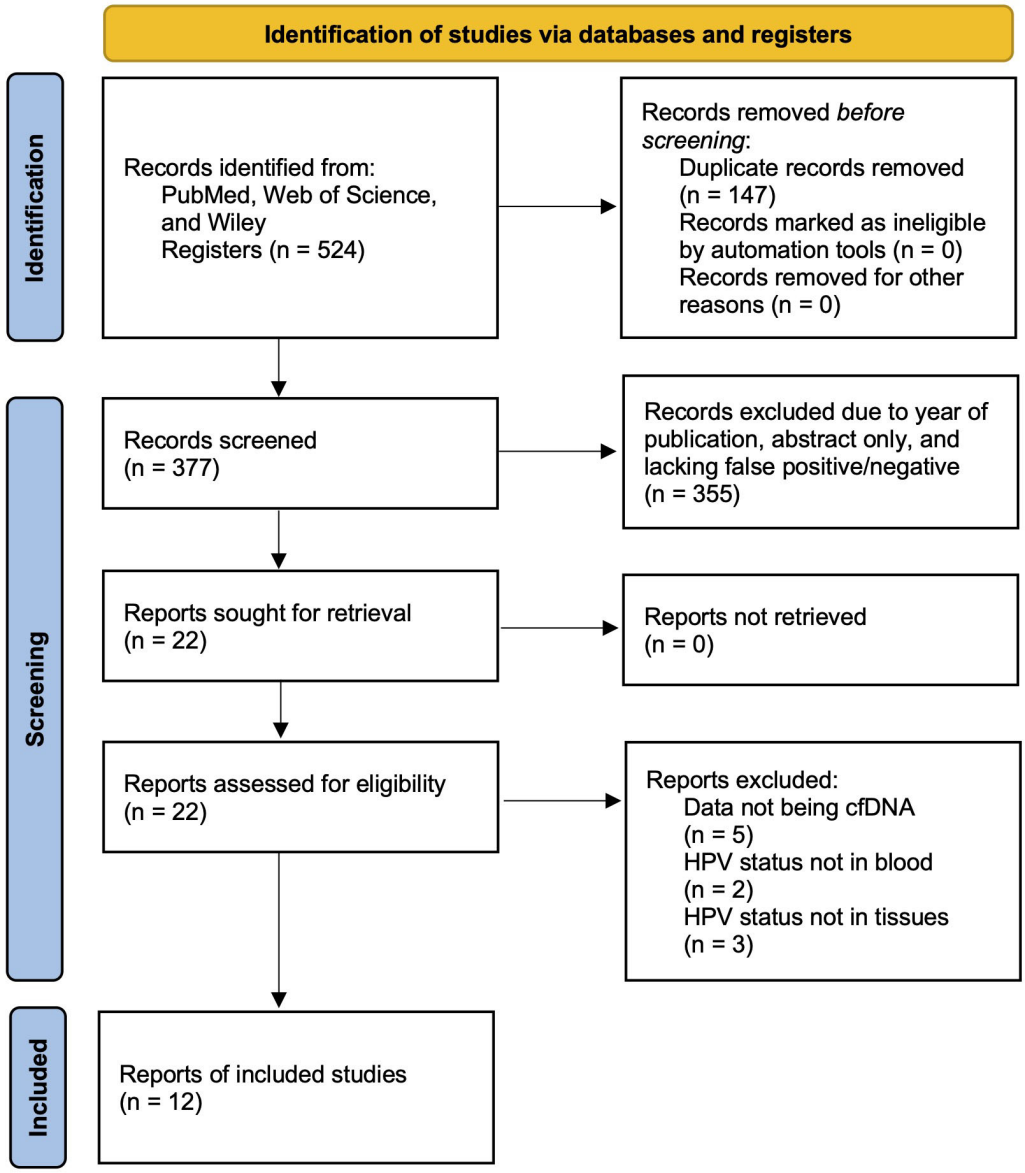
PRISMA flow chart exemplifying the study selection process included in meta-analysis and systematic review.

A systematic review and meta-analysis was performed succeeding the PRISMA (Preferred Reporting Items for Systematic Review and Meta-Analyses) guideline (44). A systematic literature search was conducted using three different databases: PubMed, Web of Science, and Wiley. This literature search was intended to identify all studies examining the diagnostic performance of cfHPV-DNA in prognosis of patients with HPV-associated HNSCC from the year of 2012 to 2024. To ensure relevance and maintain a sufficiently large selection pool, older studies were excluded to focus on more recent research reflecting current methodologies and clinical practices. The search terms used included “serum biomarker”, “HNSCC”, “cell-free HPV DNA”, “p16”, “blood”, and “biomarker”. The studies were inspected based upon title and abstract initially, and if appropriate, was then surveyed based on the full text. Abstracts which were identified to be duplicated were removed. A PRISMA flowchart showing the steps of identification, screening, and eligibility is shown in **Figure 1**.

### 2.2 Eligibility criteria

For this review, the eligibility criteria was designed according to the Population, Interventions, Comparators, Outcomes, and Study designs (PICOS) framework. Regarding the population, this included patients with HPV-positive HNSCC. The intervention was usage of liquid biopsy with cfHPV-DNA and p16 detection. The comparators were the patients who had a negative test result of cfHPV-DNA. The outcome was to identify the clinical utility and diagnostic value of cfHPV-DNA in detection of patients with HPV-positive HNSCC. Our selection criteria included studies that reported cfHPV-DNA in an appropriate number of human subjects (i.e., more than 5) which included true positives (TP), false positives (FP), true negatives (TN), and false negatives (FN). Conference abstracts, year of publication, studies lacking false positives and/or false negatives, and unpublished articles were excluded. Ten articles were ineligible due to multiple reasons: data did not precisely detect cfHPV-DNA, HPV status was not confirmed within the tissues, and studies were based on *in vitro* or *in vivo* animal experiments. Peer-reviewed publications that were in different languages other than English were not included.

### 2.3 Data extraction and quality assessment

A full-text search of twelve eligible articles was conducted to extract data such as the study characteristics (author’s name, year of publication), number of patients, site of cancer, HPV status of the cancer, HPV status in the blood, method of detection of viral DNA, and data used for analyses [sensitivity and specificity or true positives, false positives, false negatives, true negatives, and area under the curve (AUC) values] (45–56). Quality assessment for this diagnostic accuracy study was based on using Quality Assessment of Diagnostic Accuracy Studies QUADAS-2 (57). QUADAS-2 was applied to calculate and account for the risk of bias and quality of the twelve studies included. According to QUADAS-2 the risk of bias was rated high, low, or unclear in all the included studies. Index test, reference standard, patient selection, and flow and timing were the four main domains attempting to be fulfilled. All the domains are evaluated in terms of the risk of bias, while patient selection, index test, and reference standard are the three domains used to assess concerns regarding applicability.

### 2.4 Statistical analysis

The software STATA (v. 18.0) and Revman (v. 5.4) were used for statistical analysis (58, 59). The authors used a random-effects model to calculate the specificity, sensitivity, diagnostic odds ratios, and diagnostic likelihood ratios with pooled 95% confidence intervals. The outcome of these measurements was plotted in a Forest plot using STATA. A bivariate analysis was used to plot a hierarchical summary receiver operating characteristic curve (HSROC) which shows the overall diagnostic test accuracy (60). The HSROC curve plots true positive rate (sensitivity) against the false positive rate (specificity) and shows how these two variables vary with one another. Additionally, AUC is included in the HSROC graph which serves as a measurement of the overall quality of this diagnostic test. Cochrane’s Q test and I^2^ statistic were included in the Forest plots to evaluate statistical heterogeneity between each of the studies (61). In the I^2^ test, the values of 25%, 50%, and 75% represented low, medium, and high levels of heterogeneity. The possible presence of publication bias was determined using Deeks’ funnel plot with a p-value less than 0.05 indicating significance.

## 3 Results

### 3.1 Study selection

Using the predefined keywords and search strategy, an initial database search identified a total of 524 studies relevant to the use of cfHPV-DNA in HNSCC diagnosis. After removing 147 duplicate entries, 377 unique articles remained. A preliminary screening of titles and abstracts led to the exclusion of 355 studies based on predefined exclusion criteria. Subsequently, 22 full-text articles were assessed for eligibility. After further exclusions of 10 studies due to factors such as insufficient data, lack of proper control groups, or alternative methodologies inconsistent with the inclusion criteria, a final total of 12 studies published between 2012 and 2024 were included in this meta-analysis (**Figure 1**).

The majority of these selected studies employed droplet digital PCR (ddPCR) (50, 52, 54–56) or quantitative PCR (qPCR) (46–48, 51, 53) as their primary detection method for cfHPV-DNA in blood samples. One study (45) used a conventional PCR (cPCR) approach followed by qPCR for validation. Additionally, one study (49) implemented a next-generation sequencing (NGS) approach, further validating their findings using real-time PCR. These different detection methodologies reflect the ongoing evolution of cfHPV-DNA testing and highlight the need for standardized detection protocols in clinical practice.

### 3.2 Study characteristics

Among the 12 included studies, six different high-risk HPV genotypes were investigated, including HPV 16, 18, 31, 33, 35, and 45. Notably, all studies included HPV 16, which is the most prevalent genotype associated with HPV-driven HNSCC (62). Its dominant presence in the included studies reflects its well-documented role in the etiology of HPV-related head and neck cancers and aligns with established epidemiological data. Additionally, HPV 18 was investigated, a genotype that is more commonly linked to adenocarcinomas, with approximately 50% of HPV 18-positive cancers classified as adenocarcinomas and 12% as squamous cell carcinomas (63).

The total number of patients included across all 12 studies was 626. The cfHPV-DNA samples were collected from one of three different biofluid sources: whole blood, serum, or plasma. This variability in sample type could contribute to heterogeneity in cfHPV-DNA detection rates across studies. The sensitivity, specificity, true positive (TP), false positive (FP), false negative (FN), and true negative (TN) values for each study are presented in **Table 1**, providing a detailed comparative overview of diagnostic performance across different methodologies.

**Table 1:** Summary of the information and characteristics of the twelve included studies.

Study ID	Location	No. of patients	Age (mean)	M:F ratio	HPV studied	TP	FP	FN	TN	Sample	Method	Ref
Cao et al., 2012	USA	13	58	11:2	HPV 18	3	0	0	10	Plasma	qPCR	(45)
Ahn et al., 2014	USA	52	55	41:11	HPV 16	5	1	2	44	Plasma	qPCR	(46)
Dahlstrom et al., 2015	USA	114	60	92:22	HPV 16	5	0	9	100	Serum	qPCR	(47)
Khanal et al., 2015	USA	10	53	9:1	HPV 16, 18	6	1	2	1	Serum	qPCR	(48)
Lee et al., 2017	UK	37	61	30:7	HPV 16	1	0	0	36	Plasma	NGS	(49)
Veyer et al., 2019	France	6	56	5:1	HPV 16	1	0	0	5	Plasma	ddPCR	(54)
Chera et al., 2020	USA	114	57	84:30	HPV 16, 18, 31, 33, 35	15	0	0	99	Plasma	ddPCR	(50)
Dickinson et al., 2020	Finland	36	59	28:8	HPV 16	11	5	4	16	Serum	qPCR	(51)
Nguyen et al., 2020	Australia	35	58	29:6	HPV 16	21	7	2	5	Plasma	ddPCR	(52)
Rutkowski et al., 2020	Poland	66	60	60:6	HPV 16	5	1	0	60	Plasma	qPCR	(53)
Tanaka et al., 2021	Japan	35	62	30:5	HPV 16	6	0	3	26	Plasma	ddPCR	(55)
Siravenga et al., 2022	Italy	108	59	90:18	HPV 16, 18, 33, 35, 45	61	1	1	45	Blood	ddPCR	(56)

M:F ratio, male-to-female ratio; qPCR, quantitative PCR; ddPCR, droplet digital PCR; NGS, next-generation sequencing.

In addition to clinical and methodological parameters, demographic characteristics of the study populations were reviewed. Geographically, the studies were conducted across a diverse set of regions, including North America (USA), Europe (United Kingdom, France, Finland, Poland, Italy), Asia (Japan), and Australia (**Table 1**). This distribution reflects a relatively broad international representation, though the majority of data originate from high-income countries. The mean age of patients across the included studies ranged from 53 to 62 years, with most studies reporting an average patient age in the mid-to-late 50s. This is consistent with the known epidemiology of HPV-positive oropharyngeal squamous cell carcinoma, which tends to affect middle-aged adults. Regarding sex distribution, all studies reported a strong male predominance. Male-to-female ratios ranged from approximately 5:1 to as high as 10:1, reflecting the recognized higher incidence of HPV-positive HNSCC among men. These demographic patterns are important when interpreting the pooled diagnostic performance and suggest that further studies in more diverse and representative populations, including younger patients, females, and non-Western regions, are warranted.

### 3.3 Quality assessment

**Figure 2:**
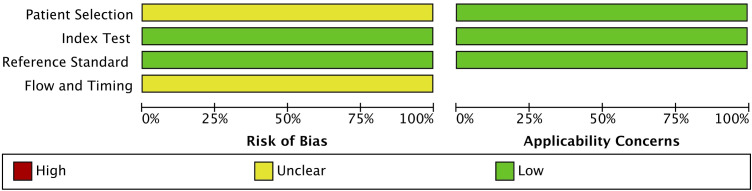
QUADAS-2 quality assessment and risk of bias and applicability concerns of the 12 included studies. Review of authors’ judgement about each domain presented as percentages across included studies.

The quality of the included studies was assessed using the QUADAS-2 tool, a validated method for evaluating diagnostic accuracy studies (**Figure 2**). All twelve studies followed a case-control study design. The overall risk of bias was low in the domains of the index test and reference standard. However, uncertainty remained regarding patient selection and flow and timing, which could influence the applicability of the findings. While these limitations are inherent in many retrospective studies, the overall methodological quality of the selected studies was deemed acceptable for inclusion in this meta-analysis.

### 3.4 Meta-analysis

**Figure 3:**
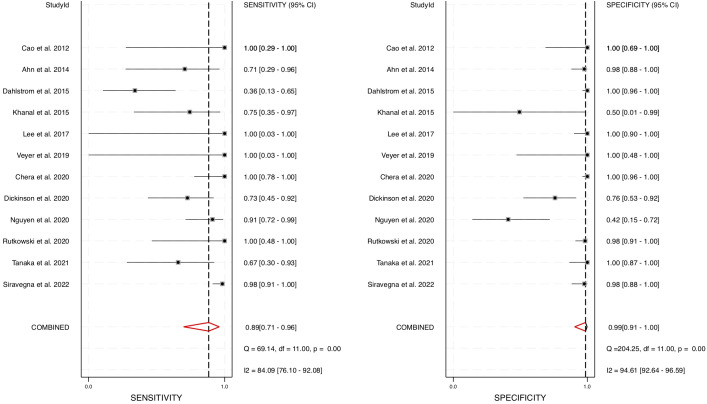
Diagnostic performance of blood cfHPV-DNA. A Forest plot with pooled sensitivity and specificity of cfHPV-DNA for diagnosis of HNSCC.

A meta-analysis was conducted using STATA software, generating a forest plot and a hierarchical summary receiver operating characteristic (HSROC) curve to evaluate the pooled sensitivity and specificity of cfHPV-DNA as a diagnostic biomarker. The pooled sensitivity for cfHPV-DNA detection in blood was determined to be 0.89 (95% CI: 0.71 – 0.96), while the pooled specificity was 0.99 (95% CI: 0.91 – 1.00) (**Figure 3**). These results indicate a strong diagnostic accuracy in distinguishing HPV-positive HNSCC from HPV-negative cases.

**Figure 4:**
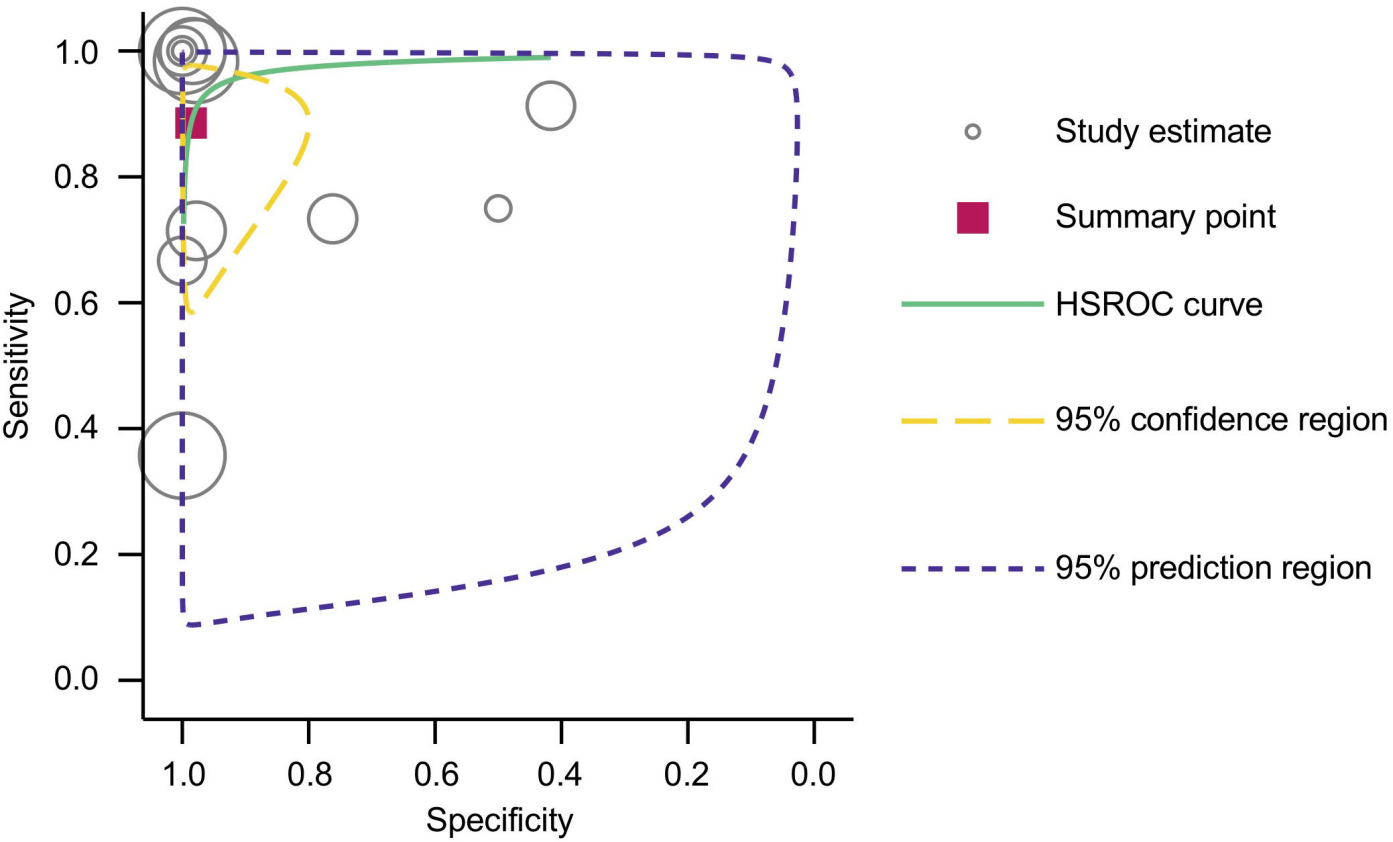
HSROC curve with pooled estimates of sensitivity and specificity of cfHPV-DNA in detection of HNSCC.

**Figure 5:**
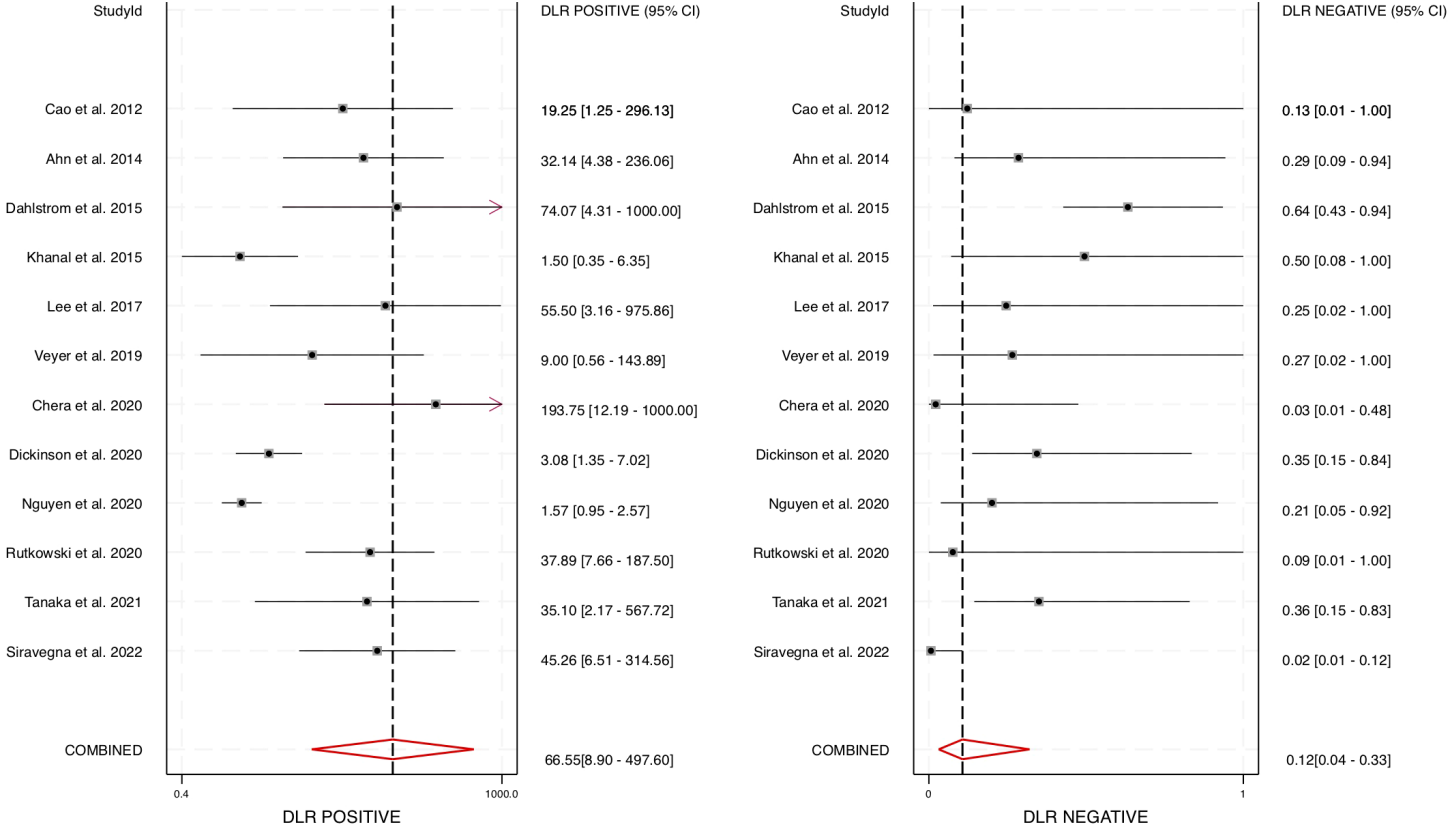
Diagnostic accuracy of cfHPV-DNA portrayed by Forest plots estimating the positive and negative DLR (diagnostic likelihood ratio).

Furthermore, the area under the HSROC curve (AUC) was calculated to be 0.98 (95% CI: 0.96 – 0.99) (**Figure 4**), signifying a highly accurate diagnostic test with minimal misclassification. The diagnostic likelihood ratio (DLR) was also computed, revealing a DLR positive of 66.55 (95% CI: 8.9 – 497.6) and a DLR negative of 0.12 (95% CI: 0.04 – 0.33) (**Figure 5**). These values suggest that a positive cfHPV-DNA result strongly increases the probability of HPV-positive HNSCC, while a negative test result significantly reduces the likelihood of disease presence.

**Figure 6:**
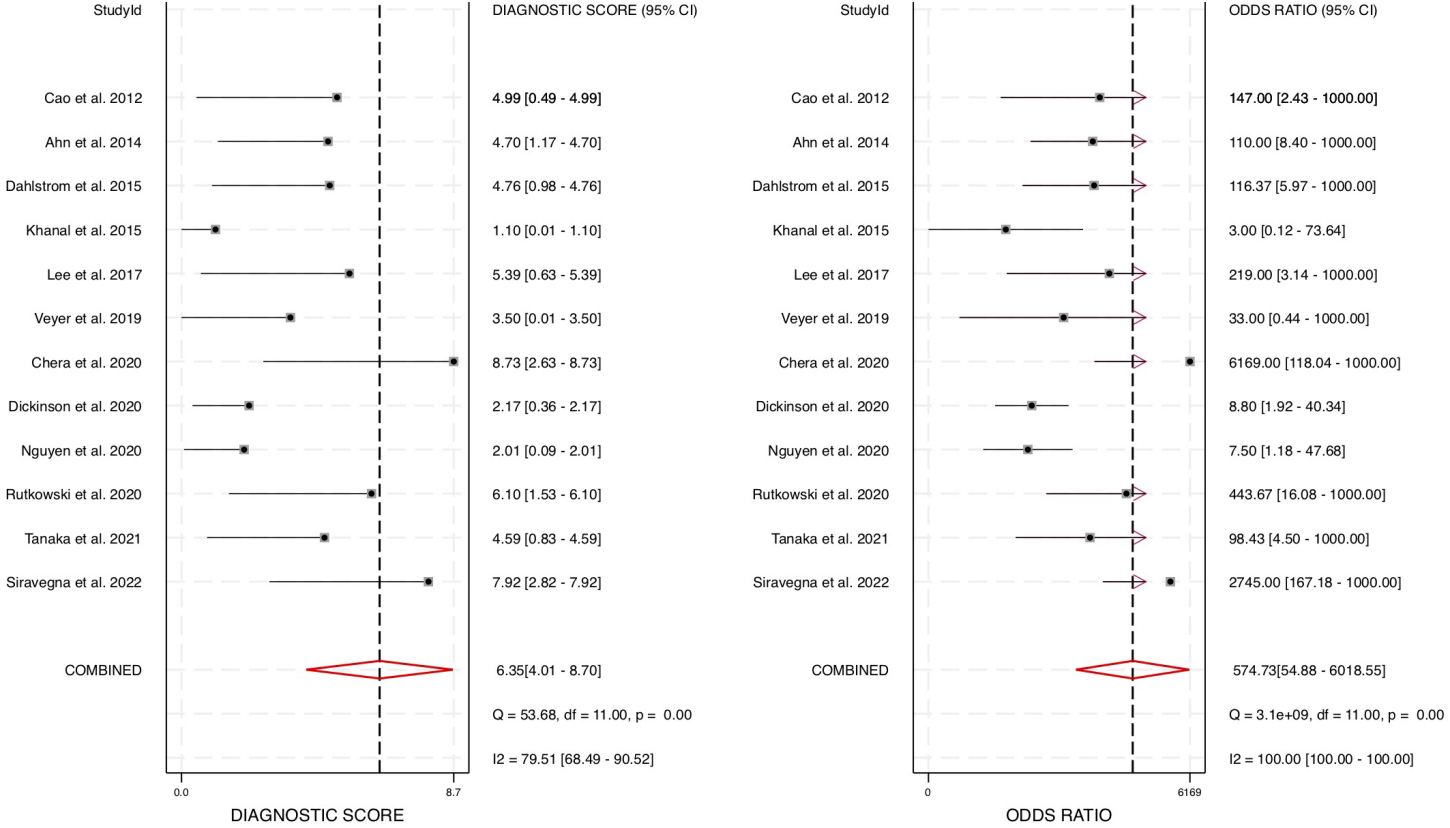
Forest plot for diagnostic odds ratio for diagnostic accuracy of cfHPV-DNA in the blood of HNSCC patients.

To further evaluate the overall diagnostic effectiveness, the pooled diagnostic odds ratio (DOR) was estimated at 574.73 (95% CI: 55 – 6019) (**Figure 6**). The DOR is a key indicator of a test’s discriminatory power, with higher values indicating stronger diagnostic capability. Given the high DOR observed, our findings strongly support the utility of cfHPV-DNA as a robust and reliable biomarker for HNSCC diagnosis.

To account for heterogeneity across the studies, we performed Cochrane’s Q test and the I² test (**Figures 3**, **6**). The presence of heterogeneity justified the use of a random-effects model, which was subsequently applied to pool the sensitivity, specificity, positive likelihood ratio, negative likelihood ratio, and AUC values across studies. The correlation coefficient for cfHPV-DNA in this meta-analysis was determined to be 0.01, further supporting its diagnostic consistency across the selected studies. Together, these comprehensive statistical analyses confirm the strong diagnostic value of cfHPV-DNA in blood for detecting HPV-driven HNSCC, reinforcing its potential for non-invasive cancer detection and monitoring.

Although this meta-analysis focused primarily on diagnostic accuracy, it is noteworthy that several included studies also reported serial cfHPV-DNA measurements during treatment. In these studies, rapid decline or complete clearance of cfHPV-DNA correlated with favorable response, while delayed or incomplete clearance was associated with disease persistence or early relapse. These findings support the potential role of cfHPV-DNA as a dynamic biomarker for treatment adaptation and reinforce its clinical relevance in the context of de-intensified therapy in HPV-positive HNSCC.

### 3.5 Publication bias

**Figure 7:**
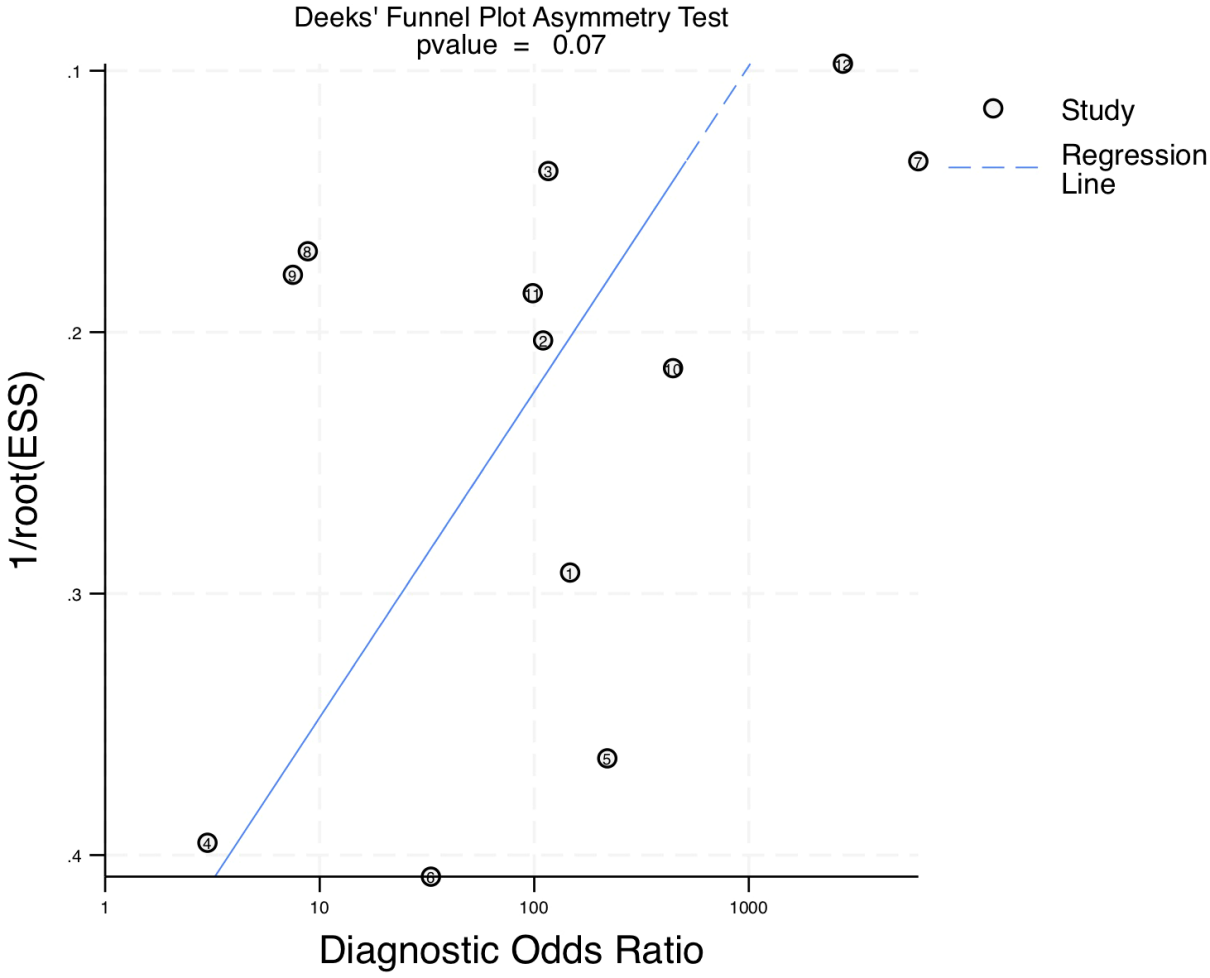
Deeks’ Funnel plot test for publication bias.

To assess potential publication bias in the included studies, Deeks’ funnel plot asymmetry test was conducted (**Figure 7**). The slope coefficient p-value was 0.07, indicating that there was no significant publication bias. The symmetrical funnel-shaped distribution of the studies further supports the reliability of the included data, suggesting that selective reporting did not substantially impact the overall findings of this meta-analysis.

## 4 Discussion

This meta-analysis provides compelling evidence supporting the diagnostic accuracy of cfHPV-DNA as a non-invasive biomarker for detecting HPV-positive HNSCC. By analyzing data from 12 studies and 626 patients, we determined that cfHPV-DNA detection in blood demonstrates a pooled sensitivity of 0.89 (95% CI: 0.71 – 0.96) and a pooled specificity of 0.99 (95% CI: 0.91 – 1.00). Furthermore, the area under the HSROC curve (AUC) was calculated to be 0.98 (95% CI: 0.96 – 0.99), indicating an exceptionally strong ability to distinguish between HPV-positive and HPV-negative cases. The high diagnostic positive likelihood ratio (DLR+) of 66.55 and the negative likelihood ratio (DLR−) of 0.12 confirm that cfHPV-DNA testing has substantial diagnostic utility, where a positive test strongly indicates disease presence, while a negative test significantly reduces the likelihood of HNSCC. Additionally, the pooled diagnostic odds ratio (DOR) of 574.73 (95% CI: 55 – 6019) further supports cfHPV-DNA as a highly effective and discriminative diagnostic tool. Together, these results confirm that cfHPV-DNA has significant diagnostic potential and could serve as a valuable addition to existing detection methods for HPV-positive HNSCC.

In HPV-associated HNSCC, the oropharynx is the most commonly affected site, accounting for the majority of cases (4–6). The oropharyngeal epithelium, particularly the lymphoid-rich tissue of the tonsils and base of the tongue, provides an ideal environment for viral entry and replication (7, 8). Combined with changes in sexual behavior, this has contributed to the rising incidence of HPV-positive oropharyngeal cancers (9–11). Unlike tumors in more accessible sites such as the oral cavity, oropharyngeal tumors often develop in anatomically concealed locations, leading to delayed clinical detection and diagnosis (34). As a result, these cancers frequently present at an advanced stage when symptoms become apparent, highlighting the need for more effective early detection strategies. cfHPV-DNA testing offers a promising non-invasive approach that could facilitate earlier identification of these tumors and improve diagnostic accuracy, particularly when used in conjunction with standard imaging techniques (56). Additionally, given that recurrence remains a major concern in HPV-positive HNSCC, a reliable surveillance method is highly desirable. Our findings suggest that cfHPV-DNA testing, with its high specificity and low false-positive rate, could serve as a valuable confirmatory tool alongside standard imaging modalities, enhancing early detection.

Beyond diagnosis, cfHPV-DNA could also play a role in clinical decision-making, particularly in treatment de-escalation strategies (42). Given that HPV-positive HNSCC patients generally have a better response to therapy and improved survival rates, there has been increasing interest in reducing treatment intensity to minimize long-term morbidity while maintaining therapeutic efficacy (29, 30). In this context, cfHPV-DNA testing could serve as a real-time, non-invasive biomarker to monitor treatment response and detect early signs of tumor regression, allowing for dynamic treatment modifications. The ability to track tumor dynamics through liquid biopsy could be particularly beneficial for patients, helping clinicians make more personalized treatment decisions. Indeed, cfHPV-DNA shows considerable promise as a tool for real-time treatment monitoring and early recurrence detection, both of which are critical in implementing treatment de-escalation strategies for HPV-positive HNSCC. Several recent studies have demonstrated that cfHPV-DNA levels decrease rapidly during chemoradiotherapy and often become undetectable upon treatment completion in patients who respond well (50, 55). Conversely, persistent or re-emergent cfHPV-DNA following treatment has been correlated with minimal residual disease and impending recurrence. This dynamic behavior allows cfHPV-DNA to serve as a surrogate marker of tumor burden and treatment response. For instance, in response-adaptive protocols, patients who achieve early clearance of cfHPV-DNA during induction chemotherapy may be eligible for radiation dose reduction or omission of concurrent chemotherapy, thereby reducing treatment-related morbidity without compromising oncologic control (42). Such applications align well with the growing paradigm of risk-adapted therapy, where biomarkers guide therapeutic intensity. In this context, cfHPV-DNA could fill a critical gap by providing a non-invasive, reproducible, and temporally sensitive indicator for tailoring treatment. The integration of cfHPV-DNA into longitudinal monitoring protocols may not only enhance surveillance but also support de-escalation decisions based on biological rather than anatomical criteria.

Despite the promising results, cfHPV-DNA testing has not yet received FDA approval for clinical use in HNSCC diagnosis, and several key challenges must be addressed before it can be integrated into routine clinical practice. One of the primary barriers is the lack of large-scale, multi-center prospective trials that comprehensively validate cfHPV-DNA’s sensitivity, specificity, and reproducibility across diverse patient populations. Although the available data strongly support its diagnostic potential, regulatory approval requires a higher level of clinical validation with standardized protocols. Another challenge is the variability in detection methodologies, as different studies have used droplet digital PCR (ddPCR), quantitative PCR (qPCR), conventional PCR (cPCR), or next-generation sequencing (NGS). These methodological differences introduce inconsistencies in cfHPV-DNA detection, emphasizing the need for a standardized, widely accepted assay with reproducible performance. Among the different detection methods used for cfHPV-DNA analysis, droplet digital PCR (ddPCR) appears to offer several advantages over other platforms. ddPCR provides absolute quantification of target DNA without the need for standard curves, offering higher sensitivity and precision compared to quantitative PCR (qPCR), which is more susceptible to variability and lower detection limits. Although qPCR is more widely available and generally less expensive, its lower sensitivity may limit its utility for detecting minimal residual disease. Next-generation sequencing (NGS) techniques offer comprehensive genomic profiling capabilities, but they are considerably more complex, time-consuming, and costly, which may limit their routine clinical use. Given these factors, ddPCR currently represents a preferable method for cfHPV-DNA detection in clinical practice due to its balance of high sensitivity, reproducibility, and moderate cost, although further standardization and validation are needed for widespread adoption.

Additionally, the clinical significance of cfHPV-DNA in guiding patient management remains to be fully established. While cfHPV-DNA shows strong diagnostic performance, it is still unclear whether it should replace or complement existing diagnostic approaches such as tissue biopsy and p16 immunohistochemistry. Furthermore, biological factors influencing cfHPV-DNA levels, such as tumor burden, clearance, and treatment effects, need to be better understood to ensure its reliability across different clinical scenarios. The half-life of cfHPV-DNA in blood is relatively short, with studies estimating it to be within several minutes to a few hours, which can affect its detectability depending on sampling time relative to tumor burden (64). Clearance of cfHPV-DNA primarily occurs through renal filtration and hepatic metabolism, while phagocytes, particularly macrophages and neutrophils, play a key role in the removal of circulating cell-free DNA via endocytosis and enzymatic digestion (65). These clearance mechanisms introduce variability in cfHPV-DNA levels, which must be accounted for when interpreting test results. Compared to well-established diagnostic tools like imaging and histopathology, cfHPV-DNA testing must demonstrate a clear advantage or equivalent accuracy in clinical utility and cost-effectiveness before widespread adoption can be considered.

A key limitation on our meta-analysis findings is the potential impact of study heterogeneity. Although a random-effects model was used to account for variations in study design, differences in sample type (blood, serum, or plasma) and cfHPV-DNA detection techniques may have contributed to result variability. Additionally, the number of eligible studies was relatively small, which could limit the generalizability of our findings. This was a direct consequence of our strict inclusion and exclusion criteria. To ensure the robustness and clinical relevance of our findings, we selected only high-quality studies that met stringent requirements, including confirmation of HPV-positive status by tissue-based methods, availability of full diagnostic accuracy data (TP, FP, TN, FN), and the use of validated cfHPV-DNA detection techniques. By applying these rigorous standards, we prioritized methodological consistency and data integrity over the size of the study pool. Nevertheless, expanding research efforts to include larger, well-controlled, prospective, multi-center studies with standardized methodologies will be essential to further validate cfHPV-DNA’s role in clinical practice.

While this meta-analysis focused on cfHPV-DNA detection in blood-based samples (plasma, serum), saliva and oral rinse have emerged as alternative, non-invasive sources for HPV detection. Compared to blood, saliva collection is easier, non-invasive, and potentially more reflective of local viral shedding in tumors of the upper aerodigestive tract. Several studies have suggested that cfHPV-DNA detection in oral fluids such as saliva or oral rinse may offer a non-invasive approach, particularly for oropharyngeal tumors, due to their anatomical proximity to the oral cavity (34, 46). Although the sensitivity of saliva-based assays has been variable and sometimes lower than that of plasma-based tests (46), these methods remain attractive due to ease of collection and patient comfort. Case reports such as Tang et al. have further highlighted their potential utility, especially in settings where blood sampling is less feasible (34). Further validation is needed to determine the optimal sampling modality for specific clinical contexts.

## 5 Conclusion and future directions

This meta-analysis demonstrates the strong diagnostic potential of cfHPV-DNA for detecting HPV-positive HNSCC and highlights its possible applications in early detection, risk stratification, and treatment de-escalation. While cfHPV-DNA testing is highly promising, further validation is needed before it can be widely adopted in clinical practice. By consolidating available data from multiple studies, our analysis strengthens the existing evidence supporting cfHPV-DNA as a diagnostic biomarker and provides a foundation for future research. To advance the clinical implementation of cfHPV-DNA testing, future efforts should focus on addressing regulatory challenges, standardizing detection assays, and conducting large-scale clinical trials. Overcoming these hurdles could enable cfHPV-DNA to become a valuable tool for early detection, treatment stratification, and long-term monitoring of HPV-positive HNSCC patients, ultimately improving patient outcomes.

## Data Availability

The original contributions presented in the study are included in the article, further inquiries can be directed to the corresponding author.
